# Ranking Nursing Diagnoses by Predictive Relevance for Intensive Care Unit Transfer Risk in Adult and Pediatric Patients: A Machine Learning Approach with Random Forest

**DOI:** 10.3390/healthcare13111339

**Published:** 2025-06-04

**Authors:** Manuele Cesare, Mario Cesare Nurchis, Nursing and Public Health Group, Gianfranco Damiani, Antonello Cocchieri

**Affiliations:** 1 Section of Hygiene, Woman and Child Health and Public Health, Gemelli IRCCS University Hospital Foundation, 00168 Rome, Italy; gianfranco.damiani@policlinicogemelli.it (G.D.); antonello.cocchieri@policlinicogemelli.it (A.C.); 2 Section of Hygiene, Department of Health Science and Public Health, Catholic University of the Sacred Heart, 00168 Rome, Italy; m.nurchis@unilink.it (M.C.N.); nursingandpublichealthgroup@gmail.com; 3 Department of Life Science, Health and Health Professions, Link Campus University, 00165 Rome, Italy

**Keywords:** nursing diagnosis, patient transfer, intensive care units, clinical deterioration, machine learning, random forest, pediatrics, adult

## Abstract

**Background/Objectives**: In hospital settings, the wide variability of acute and complex chronic conditions—among both adult and pediatric patients—requires advanced approaches to detect early signs of clinical deterioration and the risk of transfer to the intensive care unit (ICU). Nursing diagnoses (NDs), standardized representations of patient responses to actual or potential health problems, reflect nursing complexity. However, most studies have focused on the total number of NDs rather than the individual role each diagnosis may play in relation to outcomes such as ICU transfer. This study aimed to identify and rank the specific NDs most strongly associated with ICU transfers in hospitalized adult and pediatric patients. **Methods**: A retrospective, monocentric observational study was conducted using electronic health records from an Italian tertiary hospital. The dataset included 42,735 patients (40,649 adults and 2086 pediatric), and sociodemographic, clinical, and nursing data were collected. A random forest model was applied to assess the predictive relevance (i.e., variable importance) of individual NDs in relation to ICU transfers. **Results**: Among adult patients, the NDs most strongly associated with ICU transfer were *Physical mobility impairment*, *Injury risk*, *Skin integrity impairment risk*, *Acute pain,* and *Fall risk*. In the pediatric population, *Acute pain*, *Injury risk*, *Sleep pattern disturbance*, *Skin integrity impairment risk*, and *Airway clearance impairment* emerged as the NDs most frequently linked to ICU transfer. The models showed good performance and generalizability, with stable out-of-bag and validation errors across iterations. **Conclusions**: A prioritized ranking of NDs appears to be associated with ICU transfers, suggesting their potential utility as early warning indicators of clinical deterioration. Patients presenting with high-risk diagnostic profiles should be prioritized for enhanced clinical surveillance and proactive intervention, as they may represent vulnerable populations.

## 1. Introduction

Today, the wide variability in clinical conditions among both adult and pediatric patients, including acute and complex chronic conditions and the associated nursing complexity, emphasizes the need for robust approaches to identify factors able to influence clinical and organizational outcomes in hospital settings. Along with medical data, nursing diagnoses (NDs), defined as standardized clinical judgements regarding individual responses to health conditions, are central to conceptualizing nursing complexity and therefore represent the core evaluation performed by nurses based on real needs or risks [1]. NDs have long been recognized as key elements with a well-documented impact on patient outcomes (e.g., quality of life, length of hospital stays, hospital charges, and mortality) [2,3,4,5,6]. However, considering the wide range of NDs, identifying those most linked to some adverse outcomes, such as transfers to the intensive care unit (ICU), still represents a significant challenge today [3].

In this regard, studies examining the relationship between NDs and transfers to the ICU have focused on aggregated data, often assessing the total number of NDs as a predictor of clinical deterioration and confirming this relationship [3]. While this approach offers some important insights into risk prediction, it overlooks the significance of each specific ND towards this outcome. Shifting from aggregate data analysis to evaluating the individual influence of specific NDs can provide a more precise understanding of how particular patient responses to clinical conditions, represented by the single ND, affect the likelihood of transfers to the ICU.

Random forest models, proposed by Breiman [7], are promising strategies to achieve this goal, as they can capture complex associations within high-dimensional datasets [8]. These models are powerful machine learning tools, which allow for the analysis of predictors importance [9], helping to distinguish between NDs that are strongly associated with transfers to the ICU and those that play a less significant role. This statistical approach is particularly useful when datasets are populated by a large number of variables [10], such as NDs used by nurses to document care as part of a large taxonomy implemented in clinical practice [11,12]. Random forest variable importance metrics suggest that high-importance NDs could represent critical warning signs, reflecting patterns of clinical deterioration that necessitate intensive care. In contrast, less important NDs may have a weaker association with ICU transfer, potentially indicating factors with minimal weight and impact on the likelihood of admission.

Although this statistical technique could offer scientific originality when applied to NDs and risk prediction, it remains underutilized in nursing research, and its practical application is hindered by the inconsistent adoption of NDs across healthcare organizations [13]. Moreover, resistance to standardized documentation practices among nurses and healthcare systems, including the use of electronic health records (EHRs) specifically designed to collect NDs, can limit a comprehensive analysis of the role of NDs as variables implicated in ICU transfers [11,14,15]. Furthermore, today, nurses often use key physiological indicators (e.g., vital signs and laboratory results), scoring systems (e.g., Modified Early Warning Score—MEWS), or subjective clinical judgments (e.g., experience and intuition) to forecast the risk of ICU transfer [16,17]. While these approaches are useful, they may not fully capture the complexity of patient conditions, particularly when compared to data-driven models that integrate multiple variables such as clinical data and NDs. This gap highlights the potential value of advanced statistical methods and machine learning algorithms, such as random forest, in enhancing risk prediction [10], particularly regarding the risk of ICU transfer and consideration of NDs.

The aim of this study is to apply a random forest algorithm to explore the relationship between individual NDs and the risk of ICU transfer in both adult and pediatric hospitalized patients. Specifically, by assessing the individual contribution of each ND in relation to ICU transfers, this study aims to identify those most strongly associated with clinical deterioration and ICU admission. The goal is to generate a ranking of NDs based on their predictive relevance for ICU transfer risk, identifying those that may serve as early warning indicators versus those with lower predictive contribution in the study population.

## 2. Materials and Methods

### 2.1. Study Design and Setting

This retrospective, monocentric, observational study was conducted using patient data extracted from EHRs from an Italian university tertiary care hospital (with 1611 beds and 266 care units) equipped with ICUs. The dataset included clinical and nursing documentation from both adult and pediatric patients hospitalized between January and December 2022. This research was reported in accordance with the RECORD (Reporting of studies Conducted using Observational Routinely Collected Data) statement [18].

### 2.2. Study Population and Inclusion Criteria

The study population consisted of adult and pediatric patients admitted to the hospital units. Inclusion criteria encompassed all hospitalized patients aged 0 and above. Patients hospitalized for day hospital or day surgery purposes and those who did not provide consent were excluded from this study.

### 2.3. Variables

#### 2.3.1. Dependent Variable (Outcome)

The primary outcome was ICU transfer, defined as any intra-hospital transfer (either planned or unplanned) to an ICU during patient hospitalization. According to the literature, these transfers may be prompted by a variety of clinical and organizational factors (e.g., such as shortage of bed capacity, clinical deterioration, the need for patient isolation, or an increasing demand for monitoring, among others) [19,20]. Delayed ICU transfers have been associated with poorer outcomes, including prolonged length of stay and increased mortality rates [21]. As a result, hospitals aim to implement transfers quickly whenever clinical indications suggest the need for intensive care to improve the quality of care.

#### 2.3.2. Independent Variables

The independent variables were the NDs codified as “binary” (coded as 0/1, representing the absence or the presence of that ND, respectively), along with sociodemographic (age and gender) and clinical variables, such as diagnosis-related group (DRG) weight (which is a measure of medical complexity) [22] and the Elixhauser Comorbidity Index (which is a scoring system to classify comorbidities in adults, with known independent effects on patient outcomes) for adult patients [23].

### 2.4. Data Collection

Data were extracted from EHRs by the study hospitals’ Information and Communications Technology (ICT) service using standardized procedures to ensure data quality and consistency. To guarantee the alignment with the study objective, three different systems were employed, named the Professional Assessment Instrument (PAI), the Neonatal and Pediatric Professional Assessment Instrument (PAI*ped*), and the Hospital Discharge Register (HDR).

#### 2.4.1. PAI and PAIped Systems

The PAI system was used in the study hospital at the time of data collection to support the documentation of patient care according to the steps of the nursing process [24]. Utilizing a scientifically validated algorithm [25], and according to the Clinical Care Classification (CCC) System standardized taxonomy [12], the PAI system provides tailored ND suggestions based on the nursing assessment of each patient, including pediatric populations (PAI*ped*) [26]. Nurses can review, decline, or accept these suggestions, with the final plan of care incorporating all outputs generated by the system, including NDs, nursing interventions, and actions. Importantly, nurses retain the autonomy to personalize the entire plan of care, ensuring that clinical decision-making remains a fundamental component of nursing practice in the hospital where PAI or PAI*ped* are implemented. Nurses in the study hospital have been using the PAI for 12 years and the PAI*ped* for 9 years, and can therefore be considered expert users, having received ongoing institutional training on the nursing process, standardized nursing language, and nursing documentation [11].

#### 2.4.2. HDR

The HDR, representing the official medical documentation report produced by each private or public hospital in Italy, is used to collect data regarding clinical variables, hospital organization, and clinical outcomes [27]. In this study, the HDR was specifically used to identify and code the occurrence of ICU transfers within the sample, in addition to key independent variables used for the analysis (such as patient age, gender, DRG weight, and the Elixhauser Comorbidity Index). The variables collected through the HDR were integrated into the analytical model to explore their predictive contribution to ICU transfer risk alongside NDs across the considered populations.

### 2.5. Statistical Analysis

Descriptive and inferential statistics were used to analyze the data and achieve this study’s objectives. Frequencies and percentages were computed for categorical variables (such as gender and NDs), while means and standard deviations were calculated for continuous variables (including age and DRG weight). The Elixhauser Comorbidity Index was used to quantify comorbidities in adult patients. Group means were compared using a *t*-test (e.g., the mean number of NDs between adult and pediatric populations). Then, random forest models were adopted to identify the NDs that contribute the most to predicting the risk of transfer to the ICU (i.e., feature importance) in the two study populations. Each of the unique NDs was entered as an individual variable in the model, rather than being grouped into broader CCC categories (e.g., activity, coping, and health behavioral) to preserve granularity in the feature importance analysis.

The random forest methodology constitutes an ensemble learning approach that leverages multiple decision tree models in concert, thereby enhancing predictive performance and classification accuracy through collective estimation processes [28]. The data was separated into two subsets: 70% for training and 30% for testing. This approach enabled model development using the larger training portion while allowing independent validation with the previously unseen testing portion. To optimize models’ performance and ensure generalizability, random forest hyperparameters were tuned through iterative experimentation. The number of trees, maximum tree depth, and minimum number of samples required to split a node were adjusted using internal cross-validation procedures. Model validation was assessed using both out-of-bag (OOB) error and an independent validation error curve, particularly for the pediatric dataset [29]. These metrics were monitored across increasing iterations to evaluate convergence and detect potential overfitting. The alignment between the OOB error and the validation error trajectory indicated stable model behavior, supporting the reliability of the selected hyperparameter configuration [30].

All statistical tests were two-tailed, and a *p*-value < 0.05 was considered indicative of statistical significance. All analyses were performed using Stata (version 17.0) and IBM SPSS Statistics^®^ for macOS, Ver. 30 (Armonk, NY, USA: IBM Corp.).

### 2.6. Ethical Considerations

Ethical approval for this study was granted by the Institutional Review Board of the Catholic University of the Sacred Heart (Prot. ID 0012915/24, ID 6752, approved on 16 May 2024), in compliance with the Declaration of Helsinki [31] and Good Clinical Practice guidelines. Upon admission, all patients, parents, or legal guardians were informed about this study and asked to authorize the use of their data for research purposes. They were subsequently recontacted—either by telephone or email—to receive comprehensive information about the project, including its aims, the use of anonymized data, and their right to withdraw consent at any stage without consequences. If, after three contact attempts over a three-month timeframe, no response was obtained, the patient was excluded from this study. Ten trained nurses, each participating in a four-hour training session, were designated to contact participants and provide study-related information. For those who responded positively, written informed consent was formally collected. To protect privacy, the hospitals’ ICTs anonymized and coded the data following consent, prior to any analysis. Only authorized members of the research team were granted access to the anonymized dataset. In addition, healthcare professionals involved in patient documentation were also required to sign an additional informed consent form, both to authorize the scientific use of their documentation and to confirm their awareness of this study’s objectives.

## 3. Results

### 3.1. Population Characteristics

A total of 42,735 patients were included in the final analysis, comprising 40,649 adults and 2086 pediatric patients. Among the adult patients, the majority were female (60.7%); in contrast, in the pediatric group, male patients were more prevalent (56.7%). A total of 165,898 NDs were identified across the sample, corresponding to a mean of 3.88 (SD: 3.03) NDs per patient. Pediatric patients exhibited a significantly higher mean number of NDs compared to adults (4.08 vs. 3.87, respectively; F = 17.171, *p* < 0.001). Overall, 38 distinct NDs were identified in the adult population and 35 in the pediatric population. A total of 4057 patients (9.5%) were transferred to the ICU during hospitalization, with a higher proportion observed among pediatric patients compared to adults (15.8% vs. 9.2%).

Among adult patients, those transferred to the ICU were significantly older (mean age: 66.2 vs. 58.8 years, *p* < 0.001), had a higher DRG weight (median: 3.17 vs. 1.12, *p* < 0.001), a greater comorbidity burden (Elixhauser index mean: 1.02 vs. 0.59, *p* < 0.001), and a higher number of NDs (mean: 6.75 vs. 3.58, *p* < 0.001). Males were also more frequently represented in the ICU group (56.9% vs. 37.6%, *p* < 0.001).

Similarly, among pediatric patients, those transferred to the ICU were significantly younger (mean age: 5.59 vs. 8.55 years, *p* < 0.001), had a higher DRG weight (median: 2.11 vs. 0.75, *p* < 0.001), and had a greater number of NDs (mean: 6.19 vs. 3.68, *p* < 0.001). No significant gender differences were observed in this subgroup (*p* = 0.189).

A detailed overview of adult and pediatric patient characteristics stratified by ICU transfer status is presented in Table 1.

### 3.2. Feature Importance

Feature importance analysis revealed key predictors of transfer to the ICU for both adult and pediatric populations.

**Figure 1:**
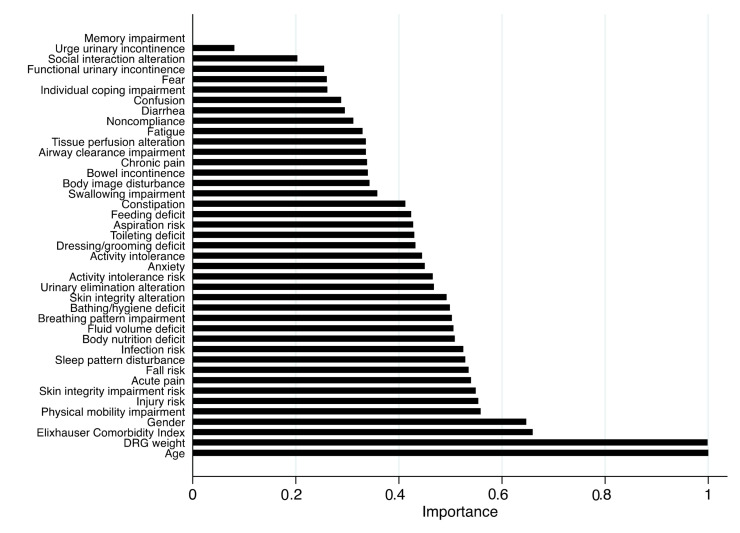
Feature importance plot from the random forest model for the adult population. Legend: DRG, diagnosis-related group. Note: Higher importance values indicate a stronger influence of the variable on ICU transfer within the model.

In adults, the most influential variables included age, DRG weight, Elixhauser Comorbidity Index, and gender, followed by NDs such as *Physical mobility impairment*, *Injury risk*, *Skin integrity impairment risk*, *Acute pain,* and *Fall risk*. The feature importance ranking, illustrated in Figure 1, highlights the relative contribution of each clinical, organizational, and nursing variables to the model’s ability to classify ICU transfer risk in adult patients (Figure 1).

**Figure 2:**
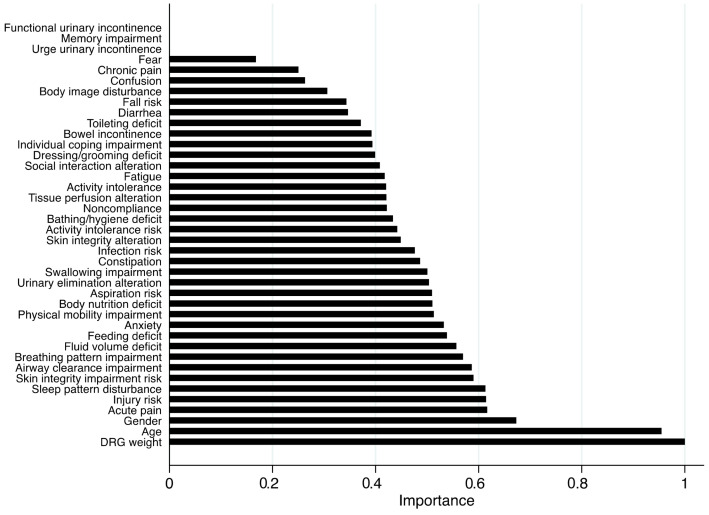
Feature importance plot from the random forest model for the pediatric population. Legend: DRG, diagnosis-related group. Note: Higher importance values indicate a stronger influence of the variable on ICU transfer within the model.

Similarly, in the pediatric cohort, DRG weight, age, and gender emerged as the most influential sociodemographic and clinical variables, while NDs such as *Acute pain, Injury risk, Sleep pattern disturbance*, *Skin integrity impairment risk*, and *Airway clearance impairment,* also contributed substantially to model performance (Figure 2).

The validation plot for the adult and pediatric model indicated stable learning dynamics, with both the OOB and validation errors decreasing and plateauing across iterations, suggesting good generalization ability and absence of overfitting (Supplementary Figures S1 and S2).

## 4. Discussion

This study applied a random forest model to identify the NDs most relevant to the risk of ICU transfer in both adult and pediatric hospitalized patients. It was important to conduct this analysis, as recognizing which specific NDs are closely linked to ICU transfer can enhance the early detection of clinical deterioration, ultimately supporting timely interventions to reduce the adverse events associated with ICU permanence [32,33,34]. While previous research has emphasized the role of aggregate counts of NDs in predicting adverse outcomes [2,3,6], this study advances the field by focusing on the relative importance of individual NDs in relation to ICU transfer. By employing a random forest model, this study harnesses the power of machine learning to detect subtle, complex profiles within clinical data, revealing which specific NDs may signal an increased risk of ICU transfer in both adult and pediatric patients.

Among adults, the most influential factors related to ICU transfer included sociodemographic and clinical variables such as age, DRG weight, Elixhauser Comorbidity Index, and gender, alongside NDs like *Physical mobility impairment*, *Injury risk*, *Skin integrity impairment risk*, *Acute pain*, and *Fall risk.* While the mentioned sociodemographic and clinical variables are well-established indicators linked to potential clinical deterioration, adverse outcomes, and increased ICU transfer risk [3,35,36,37,38,39,40], the emerging evidence on NDs underscores their value as early, nurse-identified markers of patient instability—offering a complementary perspective to traditional and common predictors.

In an effort to examine and discuss the key NDs related to ICU transfer risk, *Physical mobility impairment* is a common indicator of functional decline and is often associated with frailty, prolonged hospitalization, and increased dependency in adults [41,42]. In critically ill patients, reduced mobility can contribute to complications such as respiratory compromise, thromboembolism, or pressure injuries, all of which may necessitate ICU-level care [43,44]. Its identification by nurses may reflect a broader picture of clinical deterioration that is not always captured by physiological scores alone.

*Injury risk* also encompasses a broader set of hazards, including bleeding tendencies, confusion, and medication-related risks. This diagnosis often captures patients whose clinical status is precarious and who may rapidly decompensate, especially in the context of acute exacerbations or complex treatment regimens [45].

*Skin integrity impairment risk* is frequently associated with immobility, nutritional deficits, or hemodynamic instability [46,47]. Its presence may indicate prolonged bed rest, decreased perfusion, or underlying metabolic compromise—early warning signs that, if not addressed, can evolve into more complex conditions requiring intensive monitoring and support [48].

*Acute pain*, although often perceived as a symptom rather than a risk, seems to play a critical role in the assessment of clinical instability. Uncontrolled pain can exacerbate physiological stress, lead to respiratory compromise, impair mobility, and contribute to delirium or hemodynamic changes [49,50,51]. As such, its presence, especially when persistent or poorly managed, according to our findings, may be a red flag for impending deterioration.

*Fall risk* is another key ND that reflects underlying instability, cognitive impairment, or neuromuscular deficits—factors that often coexist with acute illness or decompensation [52,53]. Patients at high risk of falling may also be experiencing systemic weakness or hypotension, both of which may precede more severe events requiring ICU admission [54].

Similarly, in the pediatric cohort, DRG weight, age, and gender emerged as the most influential sociodemographic and clinical variables. However, a distinct ranking of NDs was identified as key factors associated with ICU transfer, including *Acute pain*, *Injury risk*, *Sleep pattern disturbance*, *Skin integrity impairment risk*, and *Airway clearance impairment*. These findings suggest that, even in this population, specific NDs can serve as early indicators of clinical deterioration by revealing underlying vulnerabilities or emerging complications.

Notably, based on our data, *Acute pain* is also among the most relevant ND associated with ICU transfer in pediatric patients. While pain in children is often underestimated or misinterpreted due to communication barriers and developmental differences, it remains a critical marker of physiological stress and clinical instability [55,56,57]. In the pediatric setting, uncontrolled or persistent pain may signal not only inadequate symptom management but also an underlying acute or evolving condition, such as infection, surgical complications, or trauma, which may precipitate the need for ICU transfer [58,59].

*Injury risk* also emerged as a key ND associated with ICU transfer in pediatric patients. As observed in the adult population, this ND may reflect conditions such as impaired mobility, altered mental status, or medication-related side effects, which increase vulnerability to falls, trauma, or other adverse events [45,60]. In pediatric patients, the presence of this ND may signal underlying instability or evolving clinical complications requiring closer monitoring or escalation to the ICU.

*Sleep pattern disturbance* emerges as a particularly relevant ND in relation to ICU admission among pediatric patients. Sleep disruption in hospitalized children has been associated with increased stress, altered immune function, and behavioral dysregulation [61,62]. It may also reflect environmental stressors, uncontrolled symptoms (such as pain or dyspnea), or evolving neurological changes [61,63]. Based on our findings, when identified early, this diagnosis may serve as a subtle yet meaningful indicator of clinical decompensation or the need for increased monitoring.

*Skin integrity impairment risk* is another significant ND strongly associated with ICU transfer in pediatric patients. This ND, as evidenced in adults, often reflects factors such as immobility, nutritional deficits, moisture exposure, or medical devices, which can compromise skin barrier function [64,65]. In children, particularly those who are critically ill or dependent on medical technology, the risk of skin breakdown may indicate a broader state of physiological vulnerability and systemic deterioration requiring ICU transfer [66].

*Airway clearance impairment* also emerged as a crucial ND associated with ICU admission in pediatric patients. This condition may arise from a range of underlying factors, including acute respiratory infections, neuromuscular disorders, or congenital anomalies, that compromise airway patency and the ability to effectively clear secretions [67,68]. The identification of this ND often reflects impending or actual respiratory compromise, characterized by ineffective ventilation, hypoxemia, or increased work of breathing—well-established clinical triggers for ICU escalation in pediatric care [69].

Based on our novel findings, and from a clinical perspective, NDs may serve as meaningful early indicators of patient deterioration, particularly when integrated with systems-based assessments and technological support [3,32,70]. However, their predictive value should be interpreted with caution, as it may be significantly influenced by the inclusion of additional clinical predictors—such as vital signs, laboratory values, and early warning scores—not considered in the present model. Future studies should explore how nursing and non-nursing data can be jointly integrated and utilized to enhance real-time risk stratification and decision-making for ICU transfer. In this context, our findings could also inform the development of nurse-led decision support tools and early warning systems grounded in standardized nursing documentation.

However, several limitations must be acknowledged. First, the retrospective design of this study may affect the completeness and consistency of routinely collected data, potentially affecting the accuracy of prediction.

Second, the monocentric nature of this study may affect the generalizability of the results, particularly in settings where clinical nursing information systems such as the PAI or the PAI*ped* are not yet implemented [11]. Documentation practices, patient characteristics, and the adoption of standardized nursing taxonomies may differ across institutions and healthcare systems. Nonetheless, these findings provide a robust starting point for further investigation. As the use of electronic nursing documentation and standardized terminologies continues to expand [11], future multicenter studies are needed to externally validate the model and assess its applicability in diverse organizational contexts.

Third, although the random forest algorithm was chosen for its suitability in handling high-dimensional binary data and its interpretability in clinical settings, we recognize that the lack of comparison with other machine learning models limits the strength of our conclusions. Future studies should consider evaluating alternative algorithms—such as eXtreme Gradient Boosting (XGBoost), Support Vector Machines (SVMs), or ensemble logistic regression [71,72,73,74]—to assess whether different approaches might yield superior or more generalizable performance.

Fourth, physiological parameters such as vital signs, laboratory values, and early warning scores (e.g., MEWS)—which are commonly used in clinical decision-making for ICU transfer [75,76]—were intentionally excluded to isolate the predictive value of NDs. While this approach highlights the contribution of nursing data, it also omits well-established predictors of ICU transfer. Future models should integrate nursing, clinical, and physiological data to improve real-world applicability and predictive performance.

Fifth, and finally, some NDs included in the model—such as Physical mobility impairment (i.e., diminished ability to perform independent movement) and Fall risk (i.e., increased chance of conditions that results in falls)—may be conceptually or statistically correlated due to overlapping clinical features and shared underlying conditions that often co-occur in patients [77,78,79]. Although random forest models are generally robust to multicollinearity [80,81], such redundancy could influence the interpretation of variable importance. Future studies could consider dimensionality reduction or hierarchical modeling to address this issue. To address this issue more explicitly, future studies could apply dimensionality reduction or hierarchical modeling approaches to better manage potential collinearity and enhance model interpretability.

Future research should aim to validate these findings in a multicenter context and explore the implementation of real-time predictive analytics based on NDs within EHRs. Moreover, prospective studies could examine whether prioritizing high-importance NDs in clinical practice may improve patient outcomes or decision-making processes regarding ICU admissions.

## 5. Conclusions

This study demonstrates the utility of applying a random forest model to identify specific NDs most strongly associated with ICU transfer in adult and pediatric patients. By moving beyond aggregate counts, this analysis highlights the predictive value of individual NDs as early, nurse-identified indicators of clinical deterioration. These findings support the integration of standardized nursing data into predictive tools and decision-making frameworks, emphasizing the importance of nursing diagnostics in improving patient outcomes and anticipating critical care needs. Further multicenter and prospective studies are warranted to validate these results and explore their implementation in real-time clinical settings.

## Figures and Tables

**Table 1:** Characteristics of adult (N = 40,649) and pediatric (N = 2086) patients, stratified by intensive care unit transfer status.

Variable	Descriptive Statistics						
	**Adult Patients**						
	General population (N = 40,649)		Patients transferred to ICU (N = 3727)		Patients not transferred to ICU (N = 36,922)		*p*-Value ^a^
Age (mean; SD)	59.46	18.09	66.23	14.51	58.77	18.27	<0.001
Gender (N; %)							<0.001
Male	15,992	39.3	2122	56.9	13,870	37.6	
Female	24,657	60.7	1605	43.1	23,052	62.4	
DRG weight (median, IQR)	1.21	1	3.17	2	1.12	1	<0.001
Elixhauser Comorbidity Index (mean; SD)	0.63	0.83	1.02	0.95	0.59	1.17	<0.001
NDs (N = 157,394) (mean; SD)	3.87	3.05	6.75	3.21	3.58	2.87	<0.001
	**Pediatric Patients**						
	General population (N = 2086)		Patients transferred to ICU (N = 330)		Patients not transferred to ICU (N = 1756)		*p*-Value ^a^
Age (mean; SD)	8.08	5.89	5.59	5.84	8.55	5.78	<0.001
Gender (N; %)							0.189
Male	1183	56.7	198	60.0	985	56.1	
Female	903	43.3	132	40.0	771	43.9	
DRG weight (median, IQR)	0.79	1	2.11	1	0.75	1	<0.001
NDs (N = 8504) (mean; SD)	4.08	2.71	6.19	2.91	3.68	2.48	<0.001

Legend: SD, standard deviation; DRG, diagnosis-related group; IQR, interquartile range; NDs, nursing diagnoses. ^a^ = chi-squared test.

## Data Availability

The data presented in this study are available on request from the corresponding author due to restrictions (privacy, legal, and ethical reasons).

## Supplementary Materials

The following supporting information can be downloaded at https://www.mdpi.com/article/10.3390/healthcare13111339/s1, Figure S1: Validation plot for random forest adult model; Figure S2: Validation plot for random forest pediatric model.

## Abbreviations

The following abbreviations are used in this manuscript:

CCC	Clinical Care Classification System
DRG	Diagnosis-related Group
EHR	Electronic Health Record
HDR	Hospital Discharge Register
ICT	Information and Communications Technology Service
ICU	Intensive Care Unit
IQR	Interquartile Range
MEWS	Modified Early Warning Score
ND	Nursing Diagnosis
OOB	Out-Of-Bag
PAI	Professional Assessment Instrument
PAI*ped*	Neonatal and Pediatric Professional Assessment Instrument
SD	Standard Deviation
