# Supplementary figures and images for: Ranking Nursing Diagnoses by Predictive Relevance for Intensive Care Unit Transfer Risk in Adult and Pediatric Patients: A Machine Learning Approach with Random Forest
Source: Healthcare (Basel). 2025 Jun 4;13(11):1339. doi: 10.3390/healthcare13111339 (PMC12154488; doi:10.3390/healthcare13111339)

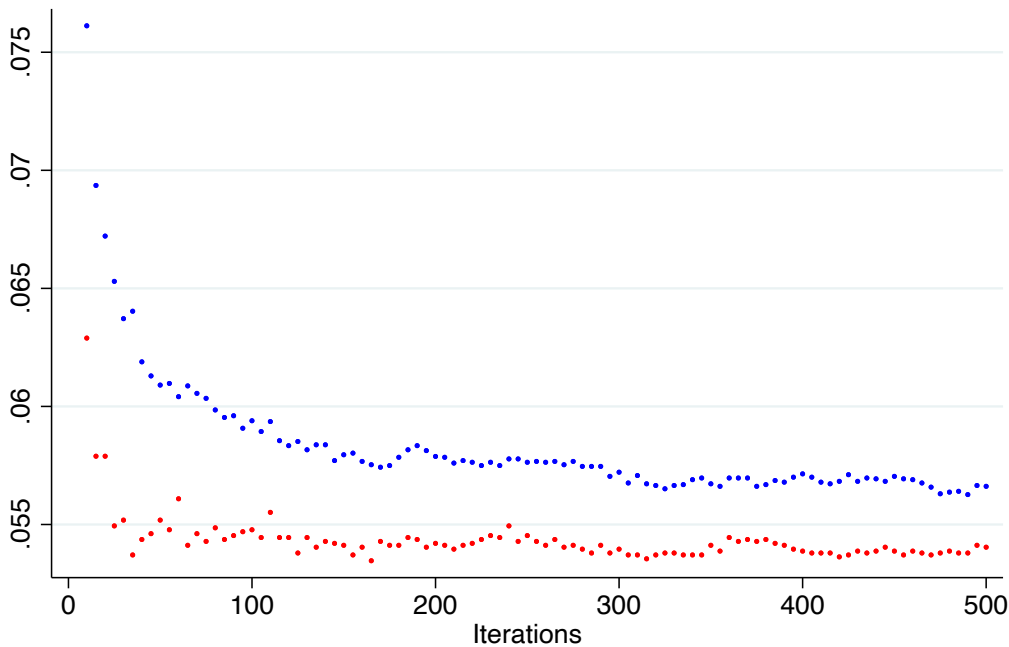

• Out-of-bag error • Validation error

Supplement: Supplementary file 1 [file healthcare-13-01339-s001.zip › Figure S1. Validation_plot_adult_patients.pdf]

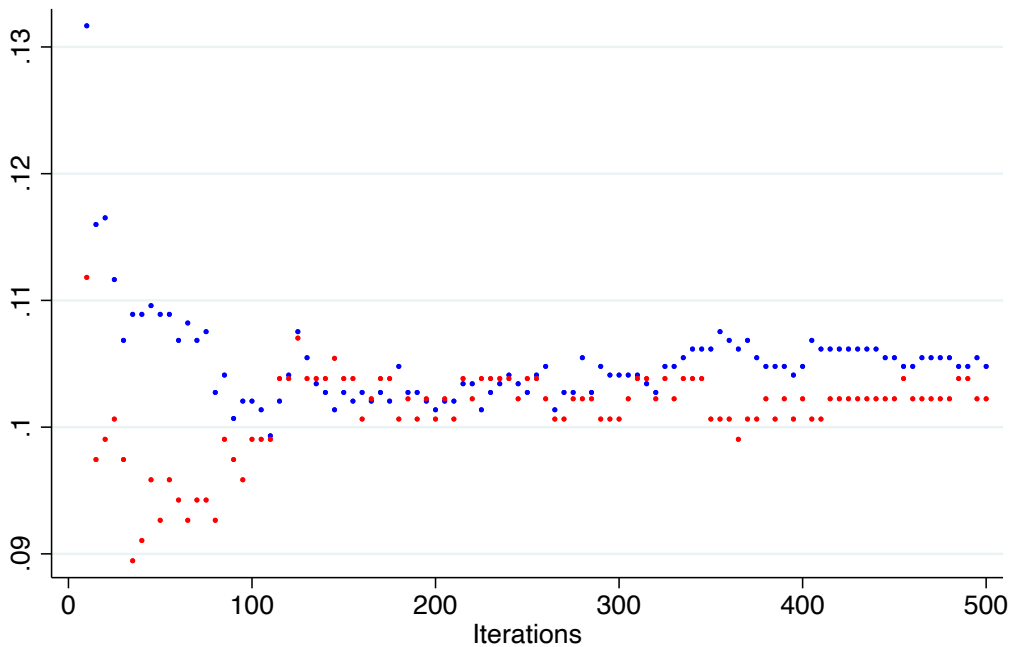

• Out-of-bag error    • Validation error

Supplement: Supplementary file 1 [file healthcare-13-01339-s001.zip › Figure S2. Validation_plot_pediatric_patients.pdf]
